# Immunonutrition for Pediatric Patients With Cow's Milk Allergy: How Early Interventions Could Impact Long-Term Outcomes

**DOI:** 10.3389/falgy.2021.676200

**Published:** 2021-07-09

**Authors:** Laura Carucci, Serena Coppola, Anna Luzzetti, Luana Voto, Veronica Giglio, Lorella Paparo, Rita Nocerino, Roberto Berni Canani

**Affiliations:** ^1^Department of Translational Medical Science, University of Naples Federico II, Naples, Italy; ^2^ImmunonutritionLab at the CEINGE Advanced Biotechnologies Research Center, University of Naples Federico II, Naples, Italy; ^3^European Laboratory for the Investigation of Food-Induced Diseases, University of Naples Federico II, Naples, Italy; ^4^Task Force for Microbiome Studies, University of Naples Federico II, Naples, Italy

**Keywords:** food allergy, gut microbiome, dietary peptides, immune tolerance, atopic march

## Abstract

Cow's milk allergy (CMA) is one of the most common food allergies and one of the main causes of food-induced anaphylaxis in the pediatric age. Moreover, up to 45% of CMA children develop other atopic manifestations later in life, a phenomenon commonly named atopic march. Thus, CMA imposes a significant cost to health care systems as well as to families, and has emerged as one of the most expensive allergic diseases. The immunonutrition strategy builds its foundation on the ability of selected dietary factors to modulate immune system development and function. Recent studies highlighted the potential of immunonutrition in the management of CMA. This review is focused on the mechanisms and long-term clinical outcomes of the immunonutrition approach in children with CMA.

## Introduction

Much has changed during the recent decades regarding prevalence, persistence, and severity of clinical features and socio-economic burden of food allergy (FA) that currently affects up to 10% of children living in Western countries (1). Based on the immune mechanisms, FA may be classified as IgE-mediated, non-IgE-mediated, or a combination of both pathways (2). In addition, children presenting with FA in early life are at increased risk of developing other allergic manifestations later in life, such as allergic asthma and rhinitis, a phenomenon commonly named atopic march (AM) (3). With an estimated prevalence of up to 3%, cow's milk allergy (CMA) is one of the most common FA and one of the main causes of food-induced anaphylaxis in the pediatric age (4). This condition imposes a significant cost to health care systems as well as to families, and has emerged as one of the most expensive allergic diseases (4). Furthermore, early life CMA could be the first step of AM, which affects up to 45% of CMA children, also after the acquisition of immune tolerance to cow's milk proteins (CMP) (5–8). Both CMA and AM derive from a negative interaction between genetic and environmental factors (3) resulting in alteration in the gut microbiome (GM) and in immune system dysfunction. These modulatory effects are at least in part mediated by epigenetic mechanisms, and are now emerging as potential targets of intervention to facilitate the immune tolerance acquisition and to limit the occurrence of AM in CMA patients (5, 9–11).

The traditional dietary management of CMA has greatly changed in the last few years, moving from a passive approach based on the strict elimination diet of CMP-containing foods, to a proactive one, able to change the CMA course (12).

The discovery of the pivotal role of selected dietary factors in influencing immune system development and function has introduced the immunonutrition concept. The application of the immunonutrition approach in the management of CMA is paving the way to “active diet therapy,” an integrated dietary strategy able to facilitate the acquisition of immune tolerance and to prevent the occurrence of AM (5, 9, 13–15).

The modern dietary management in CMA pediatric patients is focused on three targets:

- Dietary education (allergen avoidance and healthy diet)
- Ensure adequate intake of macro and micro-nutrients (stimulation of optimal body growth and development)
- Active diet therapy (stimulation of immune tolerance and protection against AM occurrence).

The immunonutrition approach could be promoted for all three of these targets. This review is focused on the objectives and long-term clinical outcomes of the immunonutrition approach in children affected by CMA.

## Dietary Education: From Allergen Avoidance to Healthy Diet Promotion

Soon after diagnosis, important information should be provided to the families for a successful elimination diet. This information includes label terminologies (including non-standard terms such as “casein” or “whey”) and possible cross-reacting foods. In addition, clear information regarding food preparation at home should be provided with the aim to limit the risk of cross-contamination (16). Table 1 displays the foods that should be avoided in designing a correct elimination diet in children affected by CMA.

**Table 1:** Labels terminology that may indicate the presence of cow's milk proteins.

Animal fat	Delactosed whey	Powdered milk
Artificial butter flavoring	Demineralized whey	Serum
Butter	Lactalbumin	Sour cream
Caramel dye	Lactalbumin-phosphate	Sour milk
Caramel flavor	Lactose	Skimmed milk powder
Casein	Milk	Whey powder
Caseinate	Milk derivatives	Whey protein concentrate
Casein rennet	Milk proteins	Whole milk powder
Cheese	Natural flavors	Yogurt
Cream	Pasteurized milk	

Considering the emerging evidence on the role of dietary factors facilitating the occurrence of FA, the modern dietary education of CMA patients should also promote a healthy diet.

Diet plays a pivotal role in influencing the composition and metabolic features of the GM, which in turn is able to directly or indirectly modulate immune system development and function introducing the concept of the “diet-GM-immune system axis” (17). The Western-type diet is made up of highly processed and synthetic foods and sugars, rich in advanced glycation end products (AGEs), and food additives (emulsifiers, sweeteners, and preservatives). The consumption of this dietary pattern has been shown to affect negatively both the GM and the immune system (18, 19). Although to date no evidence has shown a direct relation between FA and AGEs in humans, preclinical data suggest an implication of AGEs in the mechanistic onset of FA (20, 21).

On the other hand, the promotion of a healthy dietary pattern that provides a high amount of fiber, a balanced ratio of ω-6/ω-3- polyunsaturated fatty acids (PUFAs), and polyphenols able to positively modulate the GM, should be one of the nutritional strategies to beneficially impact the GM-immune system axis (22–27).

The beneficial effect of this healthy dietary pattern on the GM is due to the synergistic and interactive combinations of nutrients, with a pivotal role exerted by fiber. Microbial conversions of dietary fiber leads to the production of some immune-modulatory metabolites such as SCFAs. Our research team has recently demonstrated that the GM of children who acquired tolerance to CMP is characterized by an enrichment of bacteria producing SCFAs, including butyric acid, which can have multiple effects on the immune response through the differentiation of Treg cells and the regulation of epithelial integrity, inducing an immune tolerance status (4).

## Ensuring Adequate Body Growth and Development

Cow's milk is an important source of nutrients such as calcium, phosphorus, vitamin B2 (riboflavin), vitamin B5 (pantothenic acid), vitamin B12 (cobalamin), vitamin D, proteins, and lipids that can be deficient if cow's milk is completely excluded from the diet (28). In non-breastfed infants, the use of substitute formula is necessary until the development of immune tolerance to limit the risk of nutritional deficiencies. Commercially available substitutive formulas are commonly tested regarding allergenicity and effect on body growth pattern and are the preferred choice for optimal dietary management of CMA infants (29). The use of plant-based products, not designed for infant nutrition, should be discouraged for the high risk of nutritional deficiencies.

In the first semester of life, any calcium supplementation must be evaluated from time to time based on the composition of the substitutive formula. In the second semester, with the introduction of solid foods, the consumption of formula progressively decreases and calcium supplementation could be required when the formula intake is <500 ml/day. Moreover, in CMA children, who do not reach immune tolerance within the first year, calcium supplementation should be considered for the entire duration of the exclusion diet, based on the child's eating habits. Total calcium supplementation can vary from 600 mg/day in the early years of life to 1,000 mg/day during childhood and adolescence, according to the recommended intake for age (30). Calcium administration should always be associated with vitamin D assessment, considering a reduction from 30–40 to 10–15% in calcium absorption when vitamin D deficiency is concomitant.

Attention should be paid to the vitamin D content in substitutive formula. Vitamin D_3_ is more effective in raising serum 25(OH)D concentration than equimolar vitamin D_2_ (31), and vitamin D deficiency has been reported in CMA subjects (32, 33).

According to the international guidelines, vitamin D supplementation should be given in all CMA infants over 6 months old who receive breast milk as their main feed. In non-breastfed CMA infants, vitamin D supplementation should be evaluated based on the child's eating habits and should be continued throughout the exclusion diet period according to the recommended intakes for age, if needed (34–36). In addition to the pivotal role in calcium metabolism, vitamin D has been suggested to play a role in influencing the immune tolerance network, through a wide range of immune and non-immune mechanisms (37–39). Thus, optimal vitamin D supplementation should also be provided considering the recent evidence demonstrating an increased risk of FA development in children exposed to higher than recommended supplementation doses (40, 41).

Besides vitamin D and calcium, ω-3-PUFAs could also be relevant in the dietary management of CMA children. These micronutrients exert a wide range of beneficial immune-modulatory actions (42, 43). Deficiency of ω-3 PUFAs have been demonstrated in children affected by FA including CMA (44, 45). The origin of deficiency in these patients is still largely obscure, suggesting the importance of an individualized ω-3 PUFA supplementation in CMA children.

## Active Diet Therapy: From Substitutive Formula to Oral Immunotherapy

The concept of active diet therapy is focused on the role of varying dietary factors to treat the underlying disease, changing the disease course, and impacting symptom relief. The CMA dietary approach, which is able to facilitate immune tolerance acquisition and prevent AM occurrence in pediatric patients, perfectly matches with this concept. In this paragraph, we focus on the role of different active dietary strategies, starting from substitutive formulas, pro-, pre-, and synbiotics, to baked milk products and oral immunotherapy.

### Driving Immune System Function Through Substitutive Formulas

Whatever the clinical pattern of CMA, the mainstay of treatment is the elimination of CMP from the patient's diet. If breastfeeding is not available, the infant must be fed with a substitutive formula adapted to CMA dietary management. This formula should be adequate in terms of nutritional compounds and allergenic safety. The most used are the following: extensively hydrolyzed whey (EHWF), casein formula (EHCF), soy formula (SF), hydrolyzed rice formula (HRF), or amino acid-based formula (AAF) (46). It has been estimated that it is up to six times more expensive to feed a child with CMA, and substitutive formulas have emerged as a primary cost driver for the management of pediatric patients with CMA (46, 47). Thus, options to stimulate immune tolerance acquisition would be very welcomed by affected families and health care systems. The forefront of the immunonutrition approach is to move from a passive approach, consisting of an elimination diet to relieve symptoms, to a “pro-active” one, meaning the possibility to actively modulate the immune system toward immune tolerance.

We have recently demonstrated a different modulation on tolerogenic mechanisms elicited by the protein fraction derived from the substitutive formulas commonly used for CMA management in human cells (4). In particular, we found that only an EHCF-derived protein fraction could activate several tolerogenic mechanisms through, at least in part, an epigenetic regulation of gene expression. Other studied formulas (EHWF, HRF, SF, and AAF) were unable to modulate such mechanisms (4). Clinical trials demonstrated that EHCF could accelerate immune tolerance acquisition in CMA children if compared to other dietary strategies through, at least in part, an epigenetic modulation of the *FoxP3* gene (8, 48). CD4+/CD25+/FoxP3+ cells are central in the maintenance of immune homeostasis and tolerance. We found that only an EHCF-derived protein fraction elicited a significant activation of CD4+/CD25+/FoxP3+ Tregs, through DNA demethylation of the *FoxP3* transcription factor (4).

### Driving Immune System Function Through Pro-, Pre-, or Synbiotics

The exposure to factors that positively influence GM composition and function, such as pro- and prebiotics, could lead to a positive modulation of the immune system with final beneficial effects in children with CMA.

**Figure 1:**
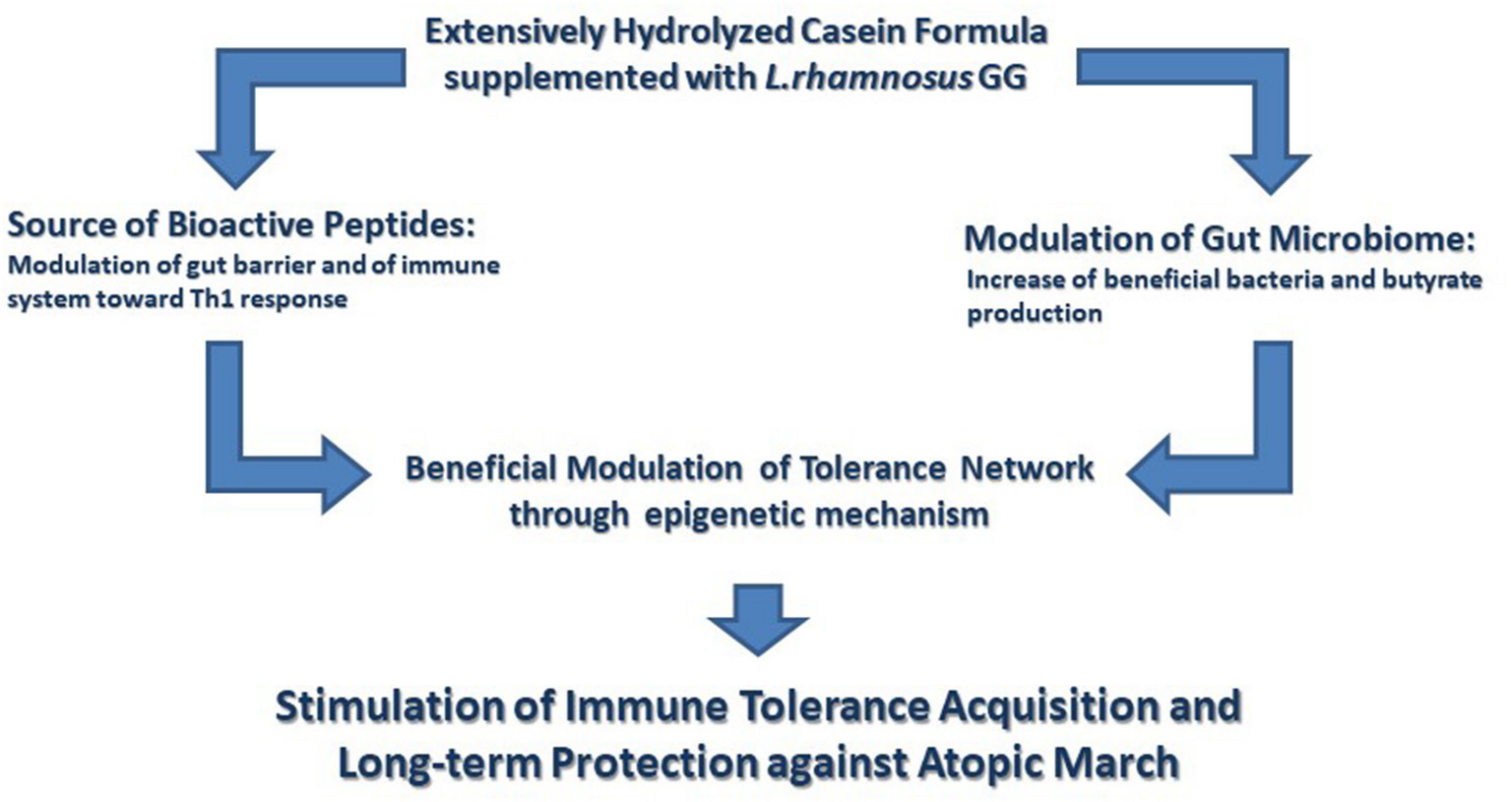
The immunomodulatory effects elicited by the extensively hydrolyzed casein formula supplemented with the probiotic *Lactobacillus rhamnosus* GG. Extensively hydrolyzed casein formula supplemented with the probiotic *Lactobacillus rhamnosus* GG (EHCF+LGG) activates several tolerogenic mechanisms. These tolerogenic mechanisms are activated by the synergist action of immunomodulatory peptides, deriving from casein hydrolysis, and by the beneficial action of LGG on gut microbiome structure and function leading to an increased production of butyrate. Many of these effects involve an epigenetic regulation of gene expression with a central role in the maintenance of immune homeostasis. Altogether these immunomodulatory effects are able to facilitate the development of immune tolerance and to reduce the occurrence of atopic march.

Probiotics are live microorganisms that confer benefits to host health when administered in adequate amounts (49). The administration of probiotics in CMA infants could be helpful in improving gastrointestinal symptoms, as demonstrated in an open randomized trial investigating the effect of *Bifidobacterium lactis* BB-12 (1 × 10^9^ CFU daily dose) and *Streptococcus thermophilus* TH-4 (1 × 10^8^ CFU daily dose) in association with a CMP elimination diet (50). More recently, a randomized, double-blind, placebo-controlled clinical trial conducted on 100 CMA infants showed the efficacy of *L. rhamnosus* (LGG) together with a CMP-free diet in improving symptoms such as bloody and mucous stool, vomiting, diarrhea, restiveness, and abdominal distension (51). The role of the probiotic LGG in hastening the development of immune tolerance in IgE-mediated CMA infants has been also demonstrated. We have shown that the administration of EHCF supplemented with LGG induced higher tolerance rate acquisition after 6 and 12 months compared with EHCF alone or with other substitutive formulas (5, 8). Moreover, at the 3-year follow-up of 220 infants with CMA, those treated with EHCF+LGG showed a greater rate of immune tolerance acquisition and a lower incidence of AM onset compared with CMA children treated with EHCF alone (6). In addition, we have shown that CMA infants treated with EHCF+LGG presented an increased number of bacteria strains able to produce the SCFA butyrate, a pivotal tolerogenic metabolite (7). Figure 1 depicts the mechanisms of action of EHCF+LGG, these immunomodulatory effects could be responsible for the beneficial action of this formula in reducing the occurrence of AM as demonstrated in a large study involving CMA children (52). These data are well in line with those of a retrospective study revealing that the first-line management of newly diagnosed CMA infants treated with EHCF+LGG may slow down AM if compared with infants treated with EHWF (53). In another double-blind controlled trial, the prenatal administration of LGG to mothers with a family history of atopic diseases, and after birth to their infants for the first 6 months, was able to protect the offspring from atopic diseases at the age of 2 (54). In line with these results, EHWF supplementation with LGG in infants with atopic eczema and CMA resulted in the affective reduction of atopic eczema and allergic symptoms. However, GM structure was unchanged in these subjects (55). Altogether, these data highlight the role of EHCF+LGG in improving clinical outcomes, in freeing up healthcare resources for alternative use, in reducing the cost of patient management, and thereby affords a cost-effective dietetic strategy in the management of CMA infants for the health care system (56, 57).

Besides the probiotics supplementation, the role of prebiotics in the GM and immune system modulation has been also suggested. Prebiotic carbohydrates are the major substrate for GM beneficial microbes' growth and/or activity. *Bifidobacterium* species can multiply in the GM of breastfed infants due to the bifidogenic effect of human milk (HM), a rich source of HM-oligosaccharides (HMOs) and of certain prebiotics (i.e., fructo- and galacto-oligosaccharides) (58). To positively modulate GM composition, it has been shown that the addition of lactose to EHWF in CMA infants is able to increase the total fecal counts of *Lactobacillus/ Bifidobacteria*. The elicited positive effect is completed by the increase of the median concentration of SCFAs, especially butyric acid, as shown by GM metabolomic analysis (59). Recently, it has been demonstrated that the co-administration of HMOs with partially hydrolyzed whey protein, can induce immunological tolerance in sensitized mice with whey and/or cholera toxin (60). In an experimental animal study, administration of a prebiotic mixture reduced the allergic skin response, whey-IgG levels, and increased the number of *Foxp3*+ cells in the proximal small intestine of whey- compared to sham-sensitized mice. Moreover, the mixture of galacto and fructo-oligosaccharides prevented the increased expression of Th2 and Th17 mRNA markers in the small intestine of the mice (61). Despite that, the ESPGHAN Committee on Nutrition concluded that there was insufficient evidence to recommend the use of prebiotics in infant formula to prevent atopic disease (62).

The definition of a synbiotic is “a mixture comprising live microorganisms and substrate(s) selectively utilized by host microorganisms that confers a health benefit to the host” (63). Recently, a double-blind, randomized, controlled multicenter trial was conducted in infants with non-IgE-mediated CMA managed with AAF containing fructo-oligosaccharides and *Bifdobacterium breve* M-16V or control product (AAF). The treatment with the synbiotic led to beneficial effects on GM composition (higher fecal percentages of Bifidobacteria and lower *Eubacterium rectale/Clostridium coccoides*) and a significantly lower use of agents for dermatological purposes and a lower incidence of ear infections in the test group compared with the control group, suggesting possible systemic immunomodulating effects elicited by this synbiotic (64). The treatment with AAF supplemented with the same synbiotic also resulted in the effective modulation of the GM of infants with CMA, bringing it close to a healthy breastfed profile (65). Despite findings that show that the use of AAF-Syn results in an improvement of GM dysbiosis, no data about an effect on immune tolerance induction has been reported. Another trial is on-going to explore the possible effect on immune tolerance in children with IgE-mediated CMA treated with AAF with or without synbiotics (scFOS/lcFOS/B breve M-16 V) (66).

The GM has a pivotal role in both innate and adaptive immune development and may have long-term effects both on the susceptibility and the persistence of allergic diseases. In ascertaining the beneficial role of AAF+synbiotics in modulating the GM and its metabolic activity, this management strategy could help in the long-term outcomes of allergic infants, such as CMP tolerance acquisition and prevention of the development of other allergic manifestations.

### Driving Immune System Function Through Baked Milk Products and Milk Ladder

When solid food introduction starts, other dietary strategies can be applied in formula- and breast-fed CMA children. The possibility to introduce processed foods containing CMP to stimulate the immune tolerance acquisition represents a novel therapeutic approach, moving from the strict elimination diet to individualized avoidance in selected patients (67).

Cow's milk contains ~3–3.5 g of proteins per 100 mL characterized by different molecular structures and thermal and digestive characteristics, resulting in different allergenic properties (68, 69). Based on their solubility at pH 4.6 and 20°C, CMP are classified into two different categories: Caseins (αS1-casein, αS2-casein, β-casein, and β-casein) are precipitating proteins and represent 80% of CMP; while the group that remain soluble are known as serum or whey proteins: ß-lactoglobulin (ß-LG), α-lactalbumin (ALA), lactoferrin (LF), bovine serum albumin (BSA), and bovine immunoglobulins (Ig), and correspond to 20% of the total CMP content (68). Caseins, ß-LG, and ALA are considered the major allergens; while LF, BSA, and Ig, although present at lower quantities, could also have a role in inducing CMA (70). Table 2 summarizes the main characteristics of CMP, based on the current studies.

**Table 2:** The main characteristics of cow's milk proteins.

**Proteins**		**Epitope**	**Heating effects on allergenicity**	**Heating in a matrix effects on allergenicity**	**Fermentation effects on allergenicity**
Caseins	αS1-casein	Sequential	Stable	Stable	Labile
	αS2-casein	Sequential	Stable	Labile	Labile
	β-casein	Sequential	Stable	Stable	Labile
	β-casein	Sequential	Stable	Labile	Stable
Whey	ß-lactoglobulin	Conformational	Labile	Labile	Labile
	α-lactalbumin bovine	Conformational	Labile	Labile	Labile
	Lactoferrin	Conformational	Labile	Labile	Labile
	Bovine serum albumin	Sequential	Stable	Labile	Labile

CMP are three-dimensional molecules held together by an electrostatic charge, and IgE binding sites, or epitopes, may be sequential or conformational. The sequential epitopes, such as αS1-casein, αS2-casein, β-casein, β-casein, and BSA, are characterized by several amino acids in a row, while the conformational epitopes, such as are ß-LG, ALA, and LF, made up of amino acids that are physically joined by the three-dimensional folding of proteins (68).

Allergenic characteristics of proteins can change during food processing such as heat treatment and lactic fermentation (71); for instance, the IgE-binding capacity can be affected by changes in the structure of proteins (aggregation, unfolding, glycation, and the occurrence of a Maillard reaction product) with a reduction of protein allergenicity (68, 70).

Moreover, CMP exposed to high temperatures in culinary recipes associated with a matrix (e.g., a muffin) showed a final reduced immunoreactivity compared with heated milk (180° C for 10 min), not included in a matrix (68, 69).

Numerous clinical studies have indicated that a large subset of children who react to unheated milk can tolerate extensively heated or fermented forms of these foods. These studies showed that among CMA children, 60–80% can eat extensively baked milk products (muffins, waffles, cakes, and breads), 60–70% can ingest fermented milk (yogurt) without presenting any adverse reactions, and about 60% can tolerate parmesan aged for 36 months (71–76).

Identifying children who tolerate baked milk products or other forms of processed milk is extremely important not only to increase the variety of diet and to improve quality of life, but also to speed up immune tolerance development (34).

Indeed, some research groups have demonstrated the role of baked milk product introduction in hastening immune tolerance acquisition to fresh milk (77, 78).

Some studies have tried to define specific IgE or skin prick test (SPT) cut-off levels to predict baked milk product reactions. For instance, Knol and colleagues proposed a specific IgE of cow's milk ≥15 KUA/L and a fresh milk SPT mean wheal diameter ≥8 mm as cut-off levels to identify a positive OFC for baked milk products in children aged ≤2 years (79). However, at present, there are no diagnostic screening tests available to identify CMA patients that are tolerant to baked products. Considering the positive effects of the introduction of these products and that more than 60% of CMA subjects tolerate baked milk products, the OFC with these processed foods should be considered in CMA children (67, 80).

**Figure 2:**
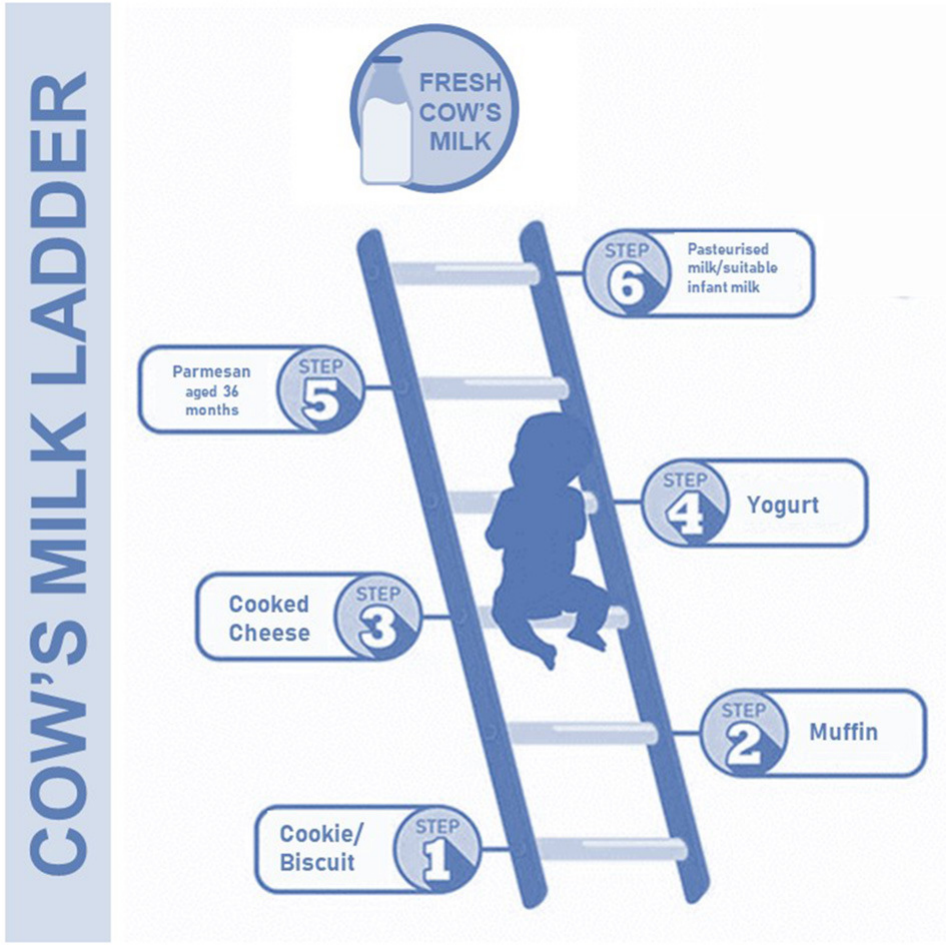
The cow's milk ladder approach. This step-by-step approach from fewer to more allergenic milk forms could speed up the immune tolerance acquisition in children with CMA.

In this way, a new approach has been designed by the British Society for Allergy and Clinical Immunology. They have proposed a “milk ladder” approach in CMA subjects to stimulate and speed up immune tolerance acquisition (34). This approach includes a step-by-step consumption of CMP, starting from fewer (baked) to more (fresh) allergenic forms of milk gradually increased at home (Figure 2). It is well known that the first home introduction of baked milk products in some CMA children could evocate an adverse reaction, and for this reason it is recommended to perform a home approach only in CMA children with a mild reaction to milk (e.g., mild rash, gastrointestinal symptoms); whereas children experiencing more severe symptoms may need undergo OFC under hospital supervision (34).

Table 3 summarizes baking temperatures and time of milk products, used in the “milk ladder” approach.

**Table 3:** Baking temperature and time of milk products used in the “milk ladder” approach.

	**Baking temperature (°F)**	**Baking time (min)**
Muffin	350	≥35
Pizza	425	≥13
Rice pudding	325	≥90
Unheated milk	None	None

Although the milk ladder was initially proposed for children with non-IgE-mediated CMA (34), several studies have shown excellent results when used in children with IgE CMA (81–83).

To explore the effects of the milk ladder approach on immune tolerance acquisition, Nowak-Wegrzyn and colleagues evaluated the effect of introducing higher doses of more allergenic forms of milk (from muffins, pizzas, rice pudding to fresh milk) vs. rigorous elimination of CMP in IgE CMA children. They found that among children who added baked milk products to their diet, 48.2% developed tolerance to fresh milk at the age of 36 months; while none of the children in the comparison arm became tolerant to baked products or fresh milk in the same time frame (82).

Similarly, Efron and colleagues showed that gradual incorporation of milk in various forms (from extensively heated and cooked milk to fresh milk) significantly reduced the time and age of IgE CMA resolution. The median age at resolution was 36 ± 3.1 months in the intervention group compared with 98± 12.3 months in the controls (CMP-free diet children) (83).

Based on these studies a safer milk ladder approach could be proposed. In detail, the first exposure of any new more allergenic food containing CMP should be performed under medical supervision and could represent a more safety strategy compared to initial home introduction (82, 83).

### Driving Immune System Function Through Oral Immunotherapy

In children with persistent IgE-mediated CMA, in which avoidance strategies are ineffective or severely limit quality of life, oral immunotherapy (OIT) could be considered as a potential active treatment. Due to the limited capacity of young children to report early allergic symptoms, and the natural tolerance acquisition in the majority of FA patients, the recommended age to start OIT is 4–5 years of age (84). OIT plays an immunological role by modulation of humoral and cell immunity. Humoral changes caused by OIT include a decrease in IgE levels and a rise in IgG levels, especially IgG4, which have a protective role on allergic reactions by blocking IgE-mediated basophil and mast cell activation. T cell response modifications include a reduction of the Th2 cell line and related cytokine expression (85, 86). These immunological changes seem to be transient, indeed OIT is also called desensitization therapy, defined as an increase in reactivity threshold to a specific allergen, that allows the patient to ingest increased amounts of a food without reaction, while continuing regular doses. The ability of OIT to induce longer-term tolerance has not yet been established (87). There are no standardized OIT protocols, but current OIT regimens typically involve the daily consumption of cow's milk, starting from a low dose, followed by incremental doses over several hours during the rush phase, and periodically (usually every 2 weeks) during the build-up phase, until the target maintenance dose is achieved. This maintenance dose is then continued daily for months to years or on-going. The main impediment to applying this approach in clinical practice is related to the high percentage of patients who cannot tolerate OIT, to the risk of severe reaction in patients with a history of anaphylaxis and to the lack of data about the ability to induce immune tolerance (87). To improve both safety and efficacy, adjunctive therapies to OIT have been evaluated in CMA children. One of the most studied adjunctive treatment is omalizumab, a humanized monoclonal anti-IgE, that seems to improve safety rather than efficacy in CMA children who underwent an OIT protocol with non-fat dry powdered milk (88). Another promising therapy is dupilumab, a monoclonal antibody directed against the alpha subunit of the IL-4 receptor (IL4R), that is able to block the IL-4 and IL-13 signaling pathways. This antibody, already approved in Italy for treatment of moderate to severe atopic dermatitis and asthma in patients aged ≥12 years of age, is now under investigation in a phase II trial (NCT04148352) as an OIT adjunctive treatment for CMA children. Data from preclinical studies on CMA mice models showed that OIT supplementation with plant-derived fructo-oligosaccharides (FOS) enhanced OIT efficacy (89) due to an increase of butyrate production, which in turn increased Treg differentiation (90). The possibility to improve OIT safety, quality of life, and the variety of diet in CMA children are the main abilities of biological therapies but data about higher efficacy compared with OIT alone require further studies.

## Discussion

Considering that CMA is one of the most prevalent and expensive allergic diseases in the pediatric age, innovative strategies to limit the disease burden are highly advocated. The immunonutrition approach could provide the answer impacting long-term outcomes, changing the CMA disease course, and may be considered the new strategy for CMA treatment in the twenty first century.

**Figure 3:**
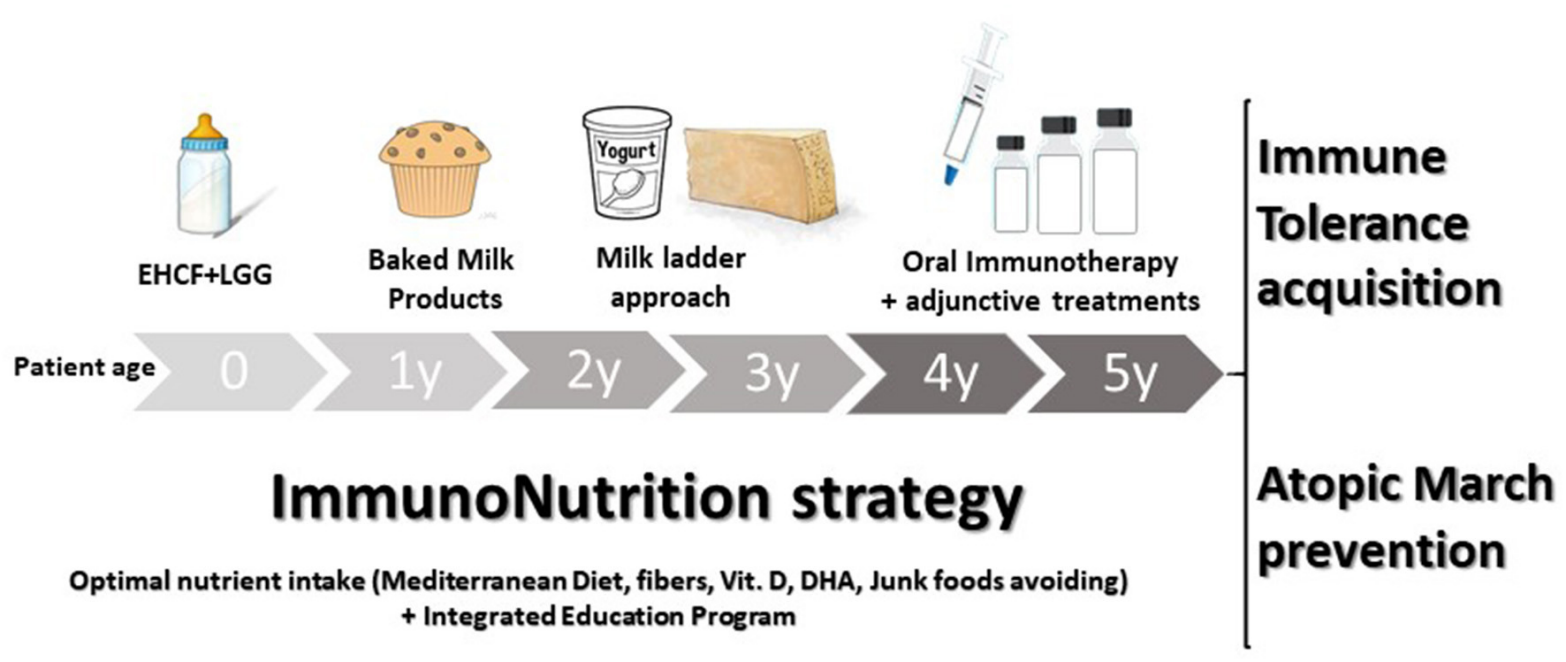
The immunonutrition strategy in cow's milk allergy (CMA) children. The active diet approach could start, in non-breastfed infants, with extensively hydrolyzed casein formula (EHCF) supplemented with the probiotic *Lactobacillus rhamnosus* GG (LGG). Then, if immune tolerance is not achieved in the first years of the disease, all the depicted steps should be considered to stimulate immune tolerance acquisition and to prevent the occurrence of other allergic manifestations. During the CMA course, the promotion of the Mediterranean diet, as well as vitamin D, calcium, and docosahexaenoic acid (DHA) supplementation should be evaluated based on the formula choice, age, and total formula daily intake. Lastly, an integrated education program aiming to limit any potential negative environmental factor exposure should be provided.

The immunonutrition strategy could be an effective approach to limit disease duration and the occurrence of AM (47). This strategy consists in an integrated multi-step approach, as illustrated in Figure 3. The first step occurs when the initial CMA diagnosis is made. Continuation of breastfeeding is highly recommended, but it is important to modulate the maternal diet avoiding CMP-containing foods and supporting an optimal adherence to a healthy dietary pattern (rich in fiber and ω-3 PUFAs). When breast milk is unavailable, the choice of a substitute formula should be based not only on allergenicity and nutritional features but also on the potential to modulate the natural disease history. Vitamin D, calcium, and ω-3 PUFAs supplementation should be considered and regularly evaluated based on the formula choice, age, and total formula daily intake. Actually, EHCF+LGG is the only substitutive formula able to stimulate immune tolerance acquisition and to prevent AM. A diet rich in fiber and ω-3 PUFAs should also be promoted after weaning together with a strict elimination of all CMP-containing foods. Then, if the child is unable to acquire immune tolerance to CMP after 12 months, baked milk products should be considered under medical supervision. If the child could tolerate baked milk products, the “milk ladder” approach could be proposed to promote both the immune tolerance acquisition and to increase the variety of the diet. In all cases, assessment of immune tolerance acquisitions every 9–12 months, depending on age and clinical history is highly recommended (91). The last but not least stage of the immunonutrition approach is OIT which could be considered in children with persistent CMA beyond the age of 4–5 years. One of the main limiting steps of OIT is the high percentage of patients who cannot tolerate it, so the adjunctive use of biologics, such as omalizumab, and improving OIT safety could be another promising strategy (84). Table 4 summarizes the key messages of the immunonutrition approach.

**Table 4:** The key messages of the immunonutrition approach in CMA children.

**In the first year of life**
A. Breastfeeding with mother following a strict CMP-free diet B. In non-breastfed infants, use extensively hydrolyzed casein formula supplemented with the probiotic Lactobacillus rhamnosus GG (EHCF + LGG) C. Dietetic assessment (evaluation of vitamin D, calcium, and ω-3 polyunsaturated fatty acids (PUFAs) supplementation)
**After 12 months**
A. OFC under medical supervision with baked milk products B. “Milk ladder” approach with less denatured CMP C. Dietetic assessment (diet rich in fiber and ω-3 PUFAs; evaluation of vitamin D, calcium, and ω-3 PUFAs supplementation)
*Assessment of immune tolerance acquisition every 9–12 months*
**From the age of 4–5**
A. Oral immunotherapy (OIT) + adjunctive treatment
*Assessment of immune tolerance acquisition every 9–12 months*

## Author Contributions

LC analyzed the literature and wrote and read the manuscript. RBC designed and structured the review and wrote and read the manuscript. SC, AL, LV, VG, LP, and RN analyzed the literature and read the manuscript. All authors listed have made a substantial, direct and intellectual contribution to the work, and approved it for publication.

## Conflict of Interest

The authors declare that the research was conducted in the absence of any commercial or financial relationships that could be construed as a potential conflict of interest.
